# Integrated transcriptome and hormone dynamics uncover biphasic defense responses in sugar beet infected by *Cercospora beticola*

**DOI:** 10.1186/s12870-026-09498-3

**Published:** 2026-07-28

**Authors:** Leonard Barnabas Ebinezer, Mari Natwick, Lorena I. Rangel, Rajdeep Jaswal, Eric Branch, Gary Secor, Melvin D. Bolton, Nathan A. Wyatt

**Affiliations:** 1 Department of Plant Pathology, Biotechnology, and Microbiology, North Dakota State University, Fargo, ND U.S.A.; 2 Sugarbeet Research Unit, Department of Agriculture- Agricultural Research Service, Fargo, ND U.S.A.; 3 Department of Plant Pathology, University of Minnesota, St. Paul, MN United States of America; 4 Present Affiliation: Department of Cell and Molecular Sciences, The James Hutton Institute, Dundee, Scotland

**Keywords:** Plant-pathogen interaction, Sugar beet, *Cercospora beticola*, Cercospora leaf spot, Transcriptomics, Phytohormones

## Abstract

**Background:**

Cercospora leaf spot (CLS), caused by the hemibiotrophic fungus *Cercospora beticola*, is a destructive foliar disease that threatens sugar beet production worldwide. Despite its economic significance, the molecular interaction between sugar beet and *C. beticola* is poorly understood.

**Results:**

We report integrated time-resolved transcriptomics and hormone profiling of CLS infection spanning the asymptomatic and symptomatic phases. Our data revealed broad transcriptional changes, with defense and hormone-related processes being most altered. We detected transcriptional signatures consistent with activation of pathogen-associated molecular pattern- and effector-triggered immunity, along with differential expression of genes encoding putative receptor-like kinases and receptor-like proteins as well as nucleotide-binding (NB) leucine‑rich repeat (LRR) receptors (NLRs). Defense activation was evident as early as 3 days post-inoculation (dpi), including upregulation of pathogenesis-related proteins, genes related to systemic acquired resistance, and the hypersensitive response (HR). At 7 dpi, several biological processes, including defense response, showed overall downregulation, suggesting a pathogen-mediated suppression of transcriptional responses. During the symptomatic phase, defense-related genes—particularly those associated with HR and numerous putative NLRs—were induced again, illustrating a bi-phasic regulation pattern. Cis-element enrichment analysis showed significant overrepresentation of defense-related WRKY and AT-hook motif nuclear-localized protein binding motifs within the protomers of genes upregulated from 11 to 25 dpi. Parallel quantification of major phytohormones demonstrated stage-dependent modulation of infection-related hormone groups across the infection cycle.

**Conclusion:**

Our analysis revealed that a susceptible sugar beet genotype mounts an early and transient defense response against *C. beticola*, which appears to be pathogen-suppressed but later reactivated during symptom development. By integrating time-resolved transcriptome analysis and hormone profiling, our study links major defense transcriptional programs and phytohormone dynamics, providing a comprehensive view of sugar beet responses to CLS that complements and extends previous genotype-focused work.

**Supplementary Information:**

The online version contains supplementary material available at https://doi.org/10.1186/s12870-026-09498-3.

## Introduction

Sugar beet (*Beta vulgaris* L.) is an economically important crop that serves as a vital source of sucrose, notably accumulating up to 20% of the root’s fresh weight [18]. Globally, Russia, Germany, France, and the United States are the top producers of sugar beet, a crop primarily cultivated in temperate regions [14]. In the United States, sugar beet accounts for more than 50% of sucrose produced, and contributes nearly 30% of global production [70]. Byproducts from sugar beet are used as fodder and feed supplements, as well as industrial feedstocks. For example, sugar beet pulp is utilized for biogas production, and beet juice and molasses can be fermented into ethanol [38].

Cercospora leaf spot (CLS), caused by *Cercospora beticola*, is the most destructive foliar disease of sugar beet worldwide [50]. Globally, CLS epidemics have resulted in large economic losses [55, 66], with reported yield losses of 30 to 50%, and in some cases, whole fields being abandoned in the absence of any management measures [50, 63]. Early sugar beet breeding programs were focused on improving concentration and extractability of sucrose with little emphasis on disease resistance [44]. Despite concerted breeding efforts for disease resistance in recent decades, CLS continues to be an important problem affecting sugar beet production.

*Cercospora beticola* is an ascomycete fungus with a hemibiotrophic lifestyle. The fungus can complete several asexual cycles within a single season under conducive conditions. The primary source of inoculum for infection is desiccation-resistant hyphal structures, known as pseudostromata, in infected plant residues. Other potential sources of inoculum include infested seed, windborne conidia, and the dispersal of *C. beticola*-infested plant material via machinery [50, 60]. After conidial germination, invasive fungal hyphae penetrate leaves through stomata [51]. The fungus then spreads through the apoplast during the asymptomatic or biotrophic phase of the infection cycle [51, 61]. Formation of characteristic necrotic lesions of CLS coincides with *C. beticola* transitioning to the necrotrophic phase.

Plants have evolved a multilayered immune network to protect themselves against pathogens [25]. Cell surface localized pattern recognition receptors (PRRs), upon detecting microbe-associated molecular patterns (MAMPs), activate the first line of defense called pattern-triggered immunity (PTI) [10]. Activation of PTI triggers a cascade of physiological defense responses to inhibit pathogen progression [10]. In response, phytopathogens secrete effectors to interfere with or suppress PTI [11, 49, 54]. These effectors can be perceived by host disease resistance proteins, triggering a quantitatively stronger and more prolonged defense response known as effector-triggered immunity (ETI) [25], culminating in localized cell death [13]. Although PTI and ETI signaling cascades have been extensively characterized in model species, the specific molecular components governing immune recognition and downstream signal transduction remain largely unresolved in sugar beet.

Despite the economic significance of CLS disease, there has been limited progress made to elucidate the molecular basis of this interaction. Previously, Weltmeier et al., [65] compared the transcriptional responses of three sugar beet genotypes (susceptible, polygenic partially resistant, and monogenic resistant) during infection with two different *C. beticola* isolates using microarrays. Large overlaps in differentially expressed genes (DEGs) identified between different genotypes during interaction with *C. beticola* were observed, albeit with differences in timing. Lou et al., [33] reported a comparative transcriptome analysis of resistant and susceptible sugar beet varieties during *C. beticola* infection at 0, 10, 20, and 30 days post inoculation (dpi). The susceptible genotype exhibited a delayed transcriptional response for defense-associated genes compared with the resistant genotype. Since differential expression was determined in comparison with the 0 dpi time point, rather than with mock-inoculated control samples collected at the respective time points, it is possible that a subset of reported DEGs may reflect variation in plant age, microbiome, and/or other abiotic parameters. Recently, in a field-based study, Jia et al., [24] compared the transcriptomes of resistant and susceptible genotypes during the peak season of CLS disease. The susceptible genotype exhibited lower expression of genes related to defense signaling, reactive oxygen species (ROS), jasmonic acid (JA) biosynthesis, and disease resistance. However, in this field-based study, the specific *C. beticola* isolate, spore load, inoculation method, and exact dpi remain unknown. Uncontrolled variables—including natural *C. beticola* diversity, co-infections, soil variations, and microbiome shifts—could significantly impact the sugar beet transcriptome. Thus, a significant knowledge gap remains regarding the nature and extent of transcriptional responses that are modulated in susceptible sugar beet during CLS infection compared to those in healthy sugar beet of the same age and environment.

Plant hormones govern a wide range of developmental programs and modulate plant adaptation to various biotic and abiotic stresses. Phytohormones such as salicylic acid (SA), JA, and ethylene (ET) are well-established regulators of plant defense pathways, and additional hormones—including auxins (IAA), abscisic acid (ABA), cytokinins (CKs), and brassinosteroids (BR)—have also been implicated in modulating plant-pathogen interactions [53]. Previously, Schmidt et al., [56] reported accumulation of ABA in *C. beticola* infected sugar beet leaves. Levels of SA and JA have been reported to decline following an increase in the early time points in susceptible genotype compared to resistant genotype [24]. However, modulation of phytohormones during asymptomatic and symptomatic phases of *C. beticola* infection in a susceptible sugar beet genotype largely remains uncharacterized.

To address these knowledge gaps, we carried out an integrated time-resolved RNA-seq analysis and hormone profiling of susceptible sugar beet host during CLS infection spanning both the asymptomatic and symptomatic phases. We present an overview of the transcriptional responses of sugar beet to *C. beticola*, highlighting the modulation of diverse classes of cell surface and intracellular receptors. By defining the temporal dynamics of various sugar beet defense responses and hormone modulation, this work establishes a framework for understanding host susceptibility and guiding future resistance research in this economically important crop.

## Materials and methods

### Plant infection and sample collection

Sporulation of *Cercospora beticola* wild-type strain 309 − 10 was induced on CV8 agar plates as previously described [57], and the harvested spores were adjusted to a concentration of 10^5^ spores ml^− 1^. Leaves of 5-week-old sugar beet plants of the susceptible variety Crystal 093 (Beta Seed, Bloomington, MN) [9] were evenly sprayed with the spore suspension or tween water. Inoculated plants were kept in a humidity chamber at 27 °C and 90% humidity for 5 days. The plants were then moved to a greenhouse with a 16-hour photoperiod and were maintained at an average temperature of 26 °C during the day and approximately 17 °C during the night with a relative humidity of 65%. For each treatment (control and inoculated) and time point (3, 7, 17, and 25 dpi), three individual leaves each from three separate plant were harvested to represent three independent biological replicates (*n* = 3). Leaves were processed individually without pooling, immediately snap-frozen in liquid nitrogen, and stored at -80 °C.

### RNA extraction and sequencing

Frozen leaf tissue was ground to powder using liquid nitrogen, and total RNA was extracted per manufacturer’s instructions using the RNeasy Plus Universal Kit (Qiagen, Germantown, MD). Total RNA was quantified using the Qubit™ RNA Broad range kit and Qubit™ fluorometer (Thermo Fisher Scientific, Waltham, MA). Purity and degradation of the RNA samples were determined using a NanoDrop Spectrophotometer 2000 (Thermo Fisher Scientific, Wilmington, DE) and Agilent 4200 TapeStation System (Santa Clara, CA), respectively. All samples had an acceptable *A*_260/280_ (> 2.0) and RIN (> 8). Library preparation, sequencing of paired-end RNA-seq libraries using Illumina HiSeq platform, and quality control were carried out by Novogene Corporation Inc. (Sacramento, CA).

### Read mapping, normalization, and statistical analysis of differential gene expression

Sequencing reads were uploaded to the SciNet USDA-ARS high-performance computing cluster, where reads were quality checked using fastQC [2], trimmed to remove adapter and poor-quality sequences with Trimmomatic using default parameters for paired end sequencing reads (ILLUMINACLIP: adapters.fa:2:30:10 LEADING:3 TRAILING:3 SLIDINGWINDOW:4:15 MINLEN:36) [6], and aligned to the sugar beet reference genome (EL10.2_2, [39]) using HISAT2 [28] with default parameters. StringTie v2.0 [45] was used to quantify transcript abundances generating gene-level count matrices using the options ‘-G’ to provide the reference annotation and ‘-e’ to limit transcript abundance quantification to only the given reference transcripts. The accessory script ‘prepDE.py3’ was then used to generate files suitable for downstream analysis [45]. Data analyses was carried out using the DESeq2 package [34] in R (www.r-project.org) by applying a design formula for differential expression that tests for effects of inoculation, applied independently to each individual time point by running default functions for estimating size factors, dispersion and negative binomial Wald test. Genes with an adjusted *p* value (Benjamini–Hochberg FDR) < 0.05 and an absolute log_2_ fold-change (log_2_FC) ≥ 1.0 (≥ 2-fold change), compared with the corresponding uninoculated control samples at each time point, were considered significantly differentially expressed.

### Bioinformatic analysis

Blast2GO analysis [21] within OmicsBox (www.biobam.com) was used to annotate differentially expressed genes (DEGs) using blastP against the UniProt database with the taxonomy filter set to *Arabidopsis thaliana* initially. For DEGs with no annotation against *A. thaliana*, blastP against the *Viridiplantae* database, a broader taxonomy group comprising all green plants, was carried out. EuKaryotic Orthologous Groups (KOG) annotation for the DEGs was obtained using eggNOG mapper (http://eggnog-mapper.embl.de/), with Viridiplantae as the taxonomic scope. The g: profiler (https://biit.cs.ut.ee/gprofiler/gost) was used for enrichment analysis of gene ontology (GO) terms of the DEGs obtained from Blast2GO analysis against a custom background consisting of all the genes detected in the RNA-seq analysis, with g: SCS significance threshold set to 0.05. Redundant GO terms were removed using REVIGO (http://revigo.irb.hr) [62]. Kyoto Encyclopedia of Genes and Genomes (KEGG) numbers obtained from eggNOG mapper annotation were used for KEGG pathway enrichment analysis using clusterProfiler R package [67] and KEGG pathway mapping. For classifying DEGs as general stress response (GSR) and core immune response (CIR), *A. thaliana* accessions reported by Bjornson et al., [5] were used as query for Blastp search in Phytozome (www.phytozome-next.jgi.doe.gov/blast-search) against the sugar beet reference genome (EL10.2_2, [39]) with a maximum expect value (E-value) significance threshold of 0.01. Variance stabilizing transformed count data was used for PERMANOVA and beta dispersion analyses using the vegan package in R. For motif enrichment analysis, 2 kbp upstream sequences of the DEGs were submitted to the Analysis of Motif Enrichment (AME) tool within the MEME suite (version 5.5.9). AME was executed using Fisher’s exact test relative to a custom background control dataset containing 2 kbp upstream sequences of all genes detected in our RNA-seq experiment with E-value < 0.01. Motifs with an adjusted *p* value < 0.01 were considered significantly enriched. Find Individual Motif Occurrences (FIMO) was utilized to scan the promoter sequences of DEGs to identify individual occurrences of these enriched motifs with a threshold of *p* value < 10^− 4^.

### Hormone analysis using liquid chromatography mass spectrometry

Phytohormones ABA, SA, JA, IAA, and ET were quantified in three biological replicates with technical triplicates from the same leaf samples from which RNA was sourced. Briefly, 100 mg of finely ground leaf tissue was spiked with deuterium-labeled internal standards (mixture of D6-ABA, D4-SA, D2-JA, and D5-IAA) and extracted using 900 µL of cold methanol: acetonitrile (50:50, v/v). Following centrifugation, the pellets were resuspended in 15% methanol. For quantification of ET, deuterium-labelled 1-aminocyclopropane-2,2,3,3-carboxylic acid (D4ACC), the precursor of ET- was spiked prior to extraction using a chilled solution of water: chloroform: methanol (3:5:12). Following a second extraction with a chilled solution of water: chloroform (3:4.5), the upper phase was dried down with speed-vac. Pellets were resuspended in 20 mM HCl solution for derivatization using AccQ-Tag chemistry per the manufacturer’s instructions (Waters, Milford, MA). Liquid chromatography (LC) separation was carried out using a ZORBAX Eclipse Plus C18 column (2.1 mm × 100 mm, Agilent Santa Clara, CA) with a flow rate of 0.45 mL/min and a gradient of the mobile phases A (0.1% formic acid) and B (0.1% formic acid/90% acetonitrile) as follows: 5% B for 1 min, to 60% B in 4 min, to 100% B in 2 min, hold at 100% B for 3 min, to 5% B in 0.5 min. Samples were analyzed using the Shimadzu Nexera 2 LC system (Columbia, MD) interfaced with a Sciex QTRAP 6500 + mass spectrometer equipped with a TurboIonSpray electrospray ion source, utilizing multiple reaction monitoring transitions optimized using standards. The instrument was set up to acquire data in positive and negative modes. Analyst software (version 1.6.3, SCIEX, Framingham, MA) was used to control data acquisition and analysis. For quantification, an external standard curve was prepared for each hormone using a series of samples containing a range of different concentrations of unlabeled hormones and fixed concentrations of a mixture of deuterium-labeled standards.

## Results

### Sugar beet extensively reprograms its transcriptome during Cercospora beticola infection

**Fig. 1:**
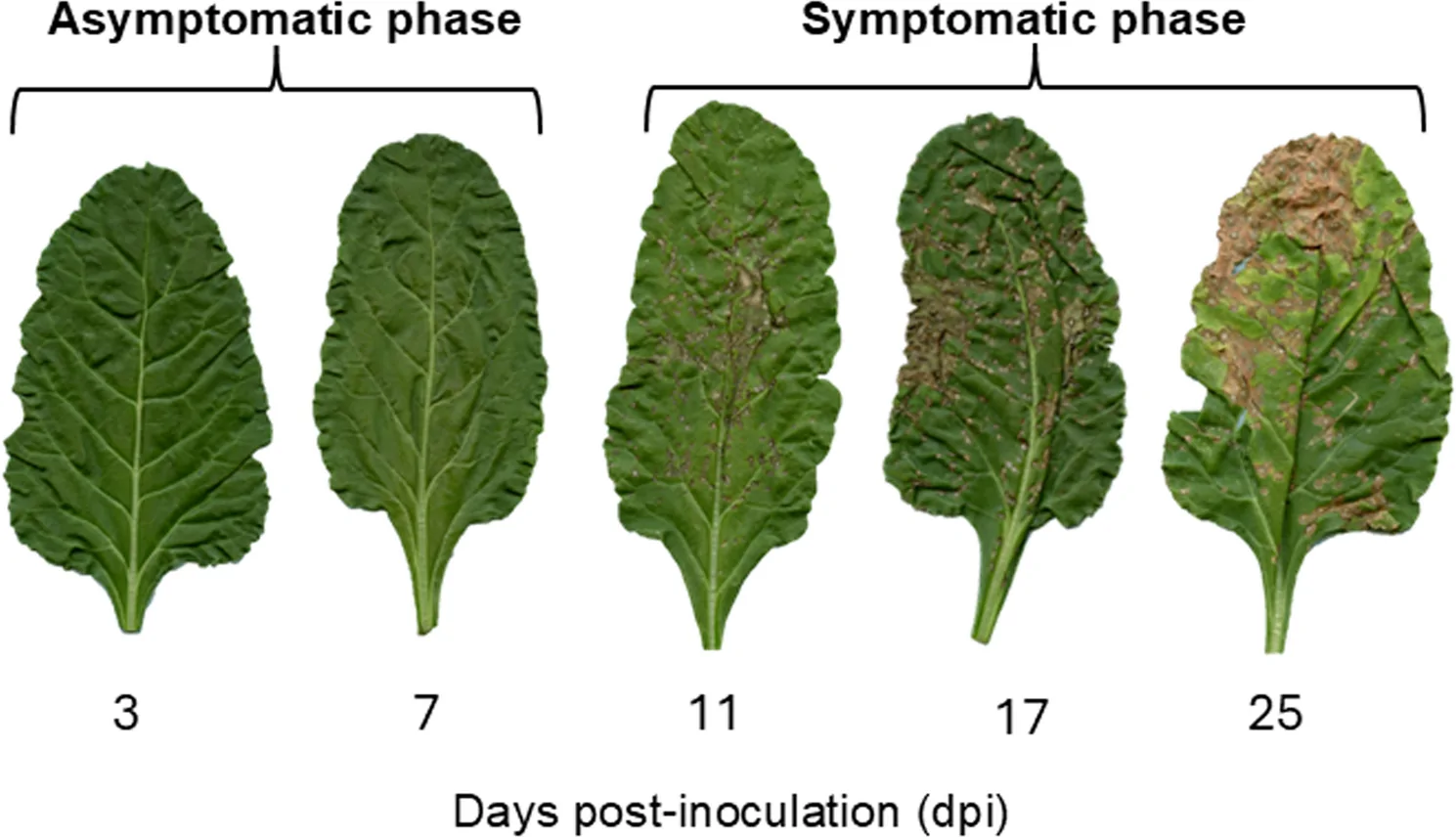
Asymptomatic and symptomatic phases of Cercospora leaf spot infection. Representative sugar beet leaves showing the transition from healthy asymptomatic leaves (3 and 7 dpi) with no visible damage to leaves showing the onset of characteristic necrotic CLS lesions (11 dpi). These lesions coalesce to form larger necrotic areas (17 dpi), ultimately resulting in completely necrotic leaves (25 dpi)

Infected leaves at 3 and 7 dpi did not have any visible symptoms and, therefore, represented the asymptomatic phase in this study (Fig. 1). Characteristic CLS symptoms began to appear at 11 dpi, and extensive lesions and necrosis were observed at 17 and 25 dpi. Hence, 11, 17, and 25 dpi represented the symptomatic phase (Fig. 1). Using Illumina sequencing of mRNA libraries, an average of 81 million reads per sample was obtained. The percentage of reads mapped to the sugar beet reference genome (EL10.2_2) [39] ranged from 64 to 99% depending on the treatment and time point of collection (Supplementary Table S1).

Principal component analysis (PCA) showed that time points representing the symptomatic phase (11, 17, 25 dpi) clustered closer, while time points representing the asymptomatic phase (3, 7 dpi) clustered farther apart from each other and the symptomatic phase time points (Supplementary Fig. S1). PERMANOVA test statistically confirmed this visual clustering (*p* = 0.001), showing that time point (dpi) accounted for 84% (R^2^ = 0.84) of the global transcriptional variance. Beta dispersion analysis verified that this grouping effect was not driven by differences in intra-group variance (*p* = 0.056) (Supplementary Table S2). PCA and PERMANOVA showed the reproducibility between biological replicates of each time point and provided evidence for distinct transcriptional responses at time points representing the asymptomatic and symptomatic phases (Supplementary Fig. S1; Supplementary Table S2).

**Fig. 2:**
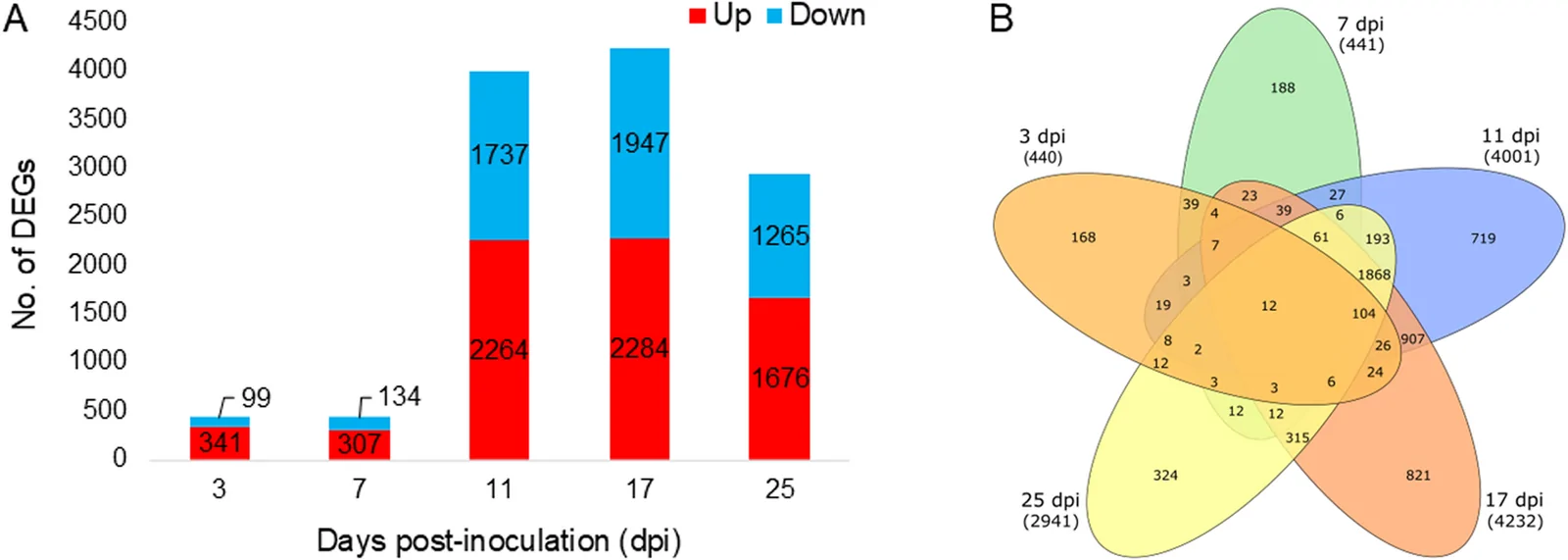
**A** Distribution of differentially expressed genes in each time point. The number of genes up- or down-regulated at each time point is denoted within the bars. **B** Comparison of DEGs between time points showing the number of DEGs unique to each infection stage and DEGs shared across infection stages

In comparison to the uninoculated control for each time point, we identified 440 and 441 DEGs at 3 and 7 dpi, respectively. At time points representing the symptomatic phase, 4,001 (11 dpi), 4,231 (17 dpi), and 2,941 (25 dpi) DEGs were identified (Fig. 2A). The majority of the DEGs were upregulated at 3 and 7 dpi, whereas later time points (11, 17, and 25 dpi) showed a more balanced distribution of up‑ and down‑regulated genes. Early stages showed limited overlap, with only 39 DEGs shared between 3 and 7 dpi, while a substantial set of 1,868 DEGs was shared among the late infection stages (11, 17, and 25 dpi). Only 12 DEGs were shared across all time points (Fig. 2B).

### Transcriptional reprogramming leads to extensive modulation of defense and hormone-related genes

**Fig. 3:**
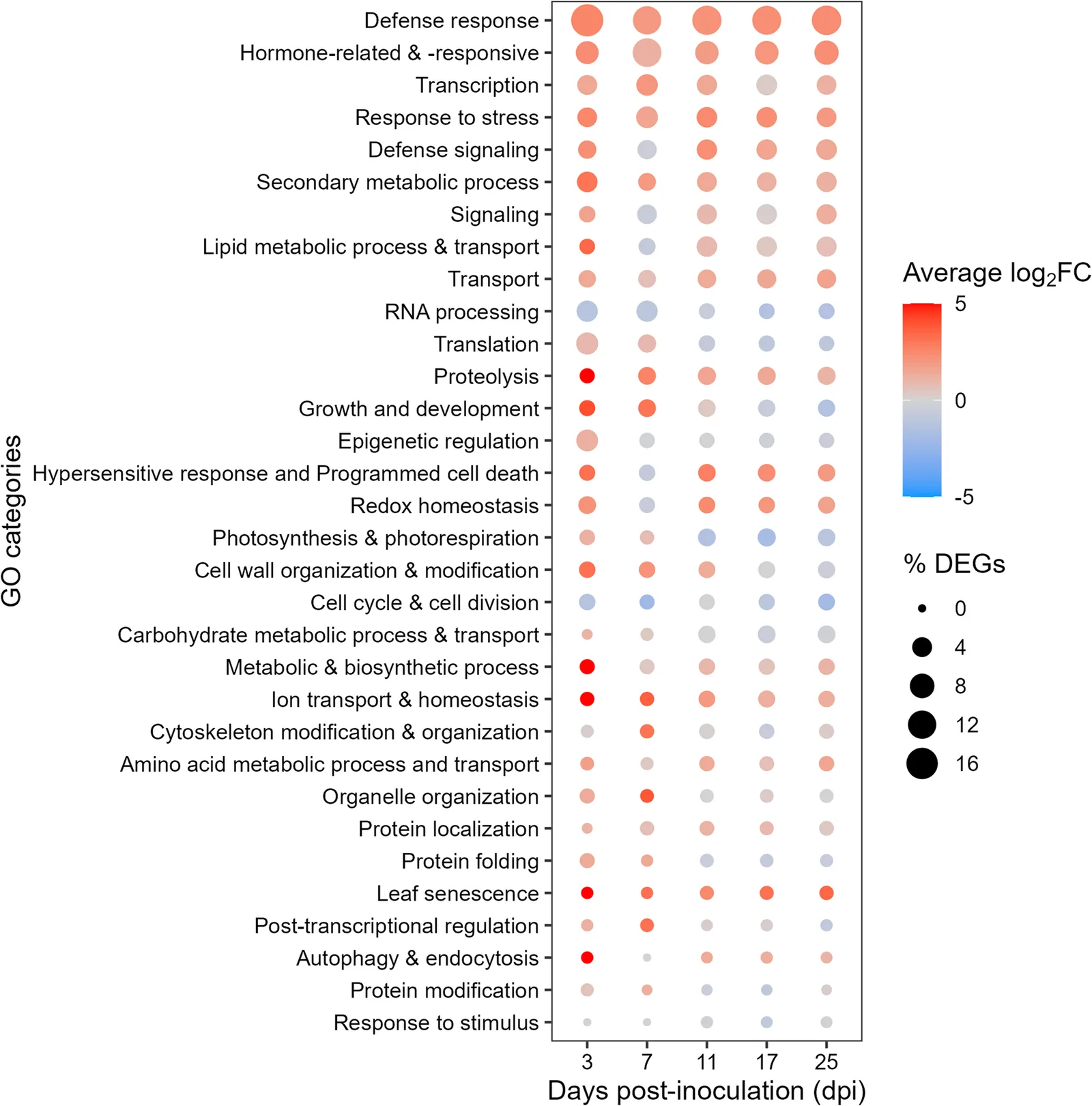
Gene ontology - based functional categorization of DEGs. DEGs are grouped into representative categories of biological processes based on the GO annotation across 3, 7, 11, 17, and 25 dpi. Node size represents the percentage of DEGs in each category, while node color indicates average log₂ fold change of each category (blue = down-regulated, red = up-regulated)

Gene ontology (GO)-based manual categorization of DEGs indicated that the highest proportion of DEGs were defense- and hormone-related responses, irrespective of the time point (Fig. 3; Supplementary Table S3). Some categories could be grouped based on their expression trend across time points using the average log_2_FC of the DEGs under each GO category. The average log_2_FC of several key functional categories related to defense, hormone, and stress-response showed an initial upregulation at 3 dpi, downregulation at 7 dpi, and a subsequent re-upregulation during 11 to 25 dpi (Supplementary Fig. S2A). We also noted that categories related to essential cellular processes and physiological functions showed a steady decline from 3 to 25 dpi (Supplementary Fig. S2B).

Consistent with functional categorization, all time points showed enrichment for one or more GO terms related to plant immunity and hormones, regardless of whether the genes were upregulated or downregulated (Supplementary Table S4). Notably, the term systemic acquired resistance (SAR) was found among the statistically significant GO terms identified in upregulated genes at 3 dpi. GO terms associated with cell death and metabolism of defense-related compounds (oxylipins and glucosamine) were predominantly represented among the downregulated genes. Terms related to the metabolism of various defense-related metabolites were identified among DEGs at 7 dpi. At 11 dpi, we observed terms related to reactive oxygen species catabolism and oxidative stress response. Several cell death-associated GO terms were represented among upregulated genes at 17 and 25 dpi. Moreover, multiple GO terms related to growth and photosynthesis were found within the downregulated genes during the symptomatic phase (Supplementary Table S4).

**Fig. 4:**
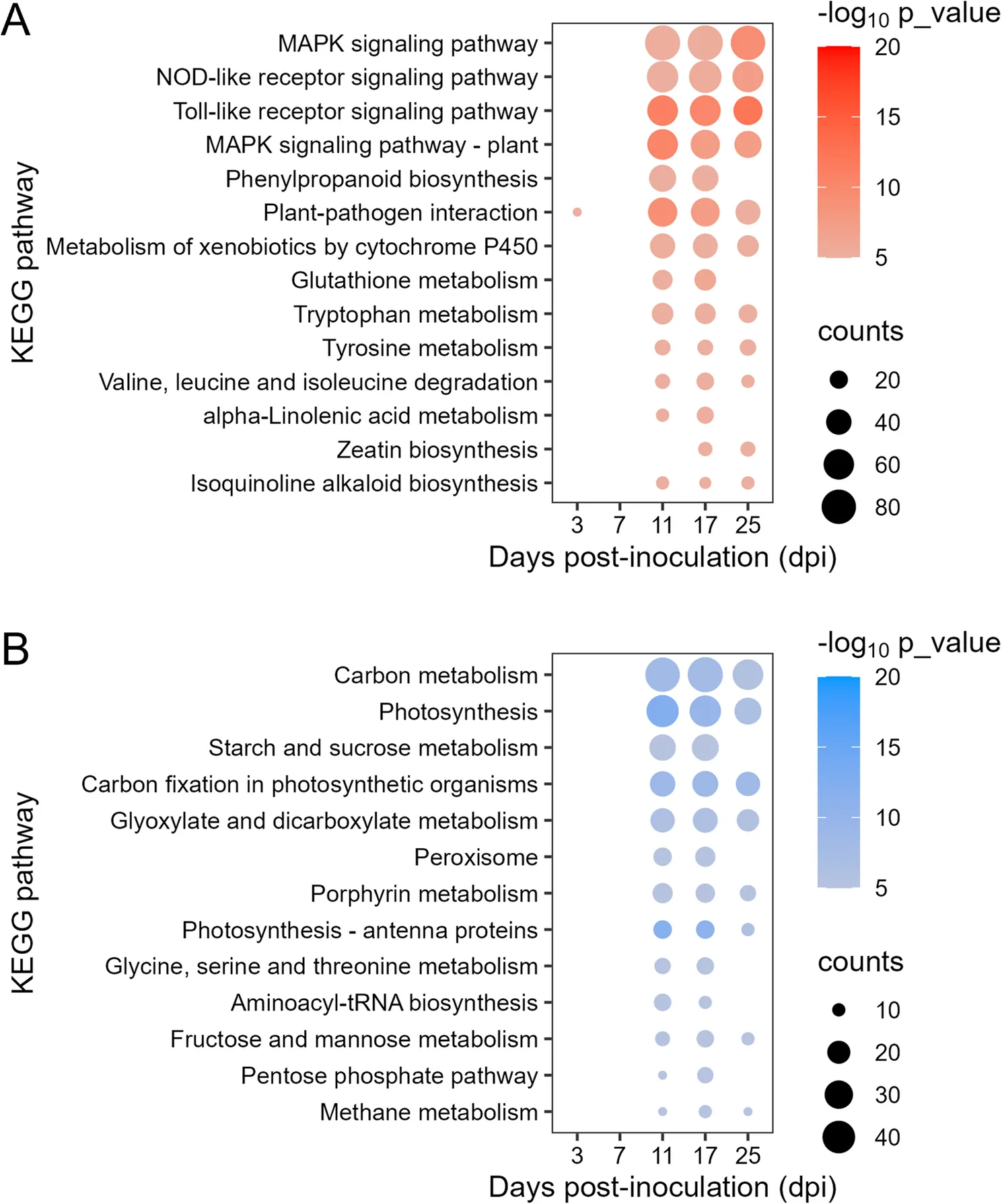
KEGG pathway enrichment analysis of up- (**A**) and down- (**B**) regulated DEGs. Node size represents the number of DEGs assigned to each pathway. Color intensity of red (upregulated) and blue (downregulated) corresponds to the statistical significance of enrichment (–log₁₀ *p* value)

Pathway enrichment analysis showed that the plant-pathogen interaction pathway was significantly enriched among the upregulated genes at all time points except 7 dpi. Multiple signaling pathways involving mitogen-activated protein kinase (MAPK), NOD-like, and Toll-like receptors were overrepresented during the symptomatic phase (Fig. 4A). While pathways associated with photosynthesis, carbon fixation, and primary metabolism were significantly enriched among the downregulated genes at the later time points (Fig. 4B).

### Key defense receptors are modulated in sugar beet during Cercospora beticola infection

**Fig. 5:**
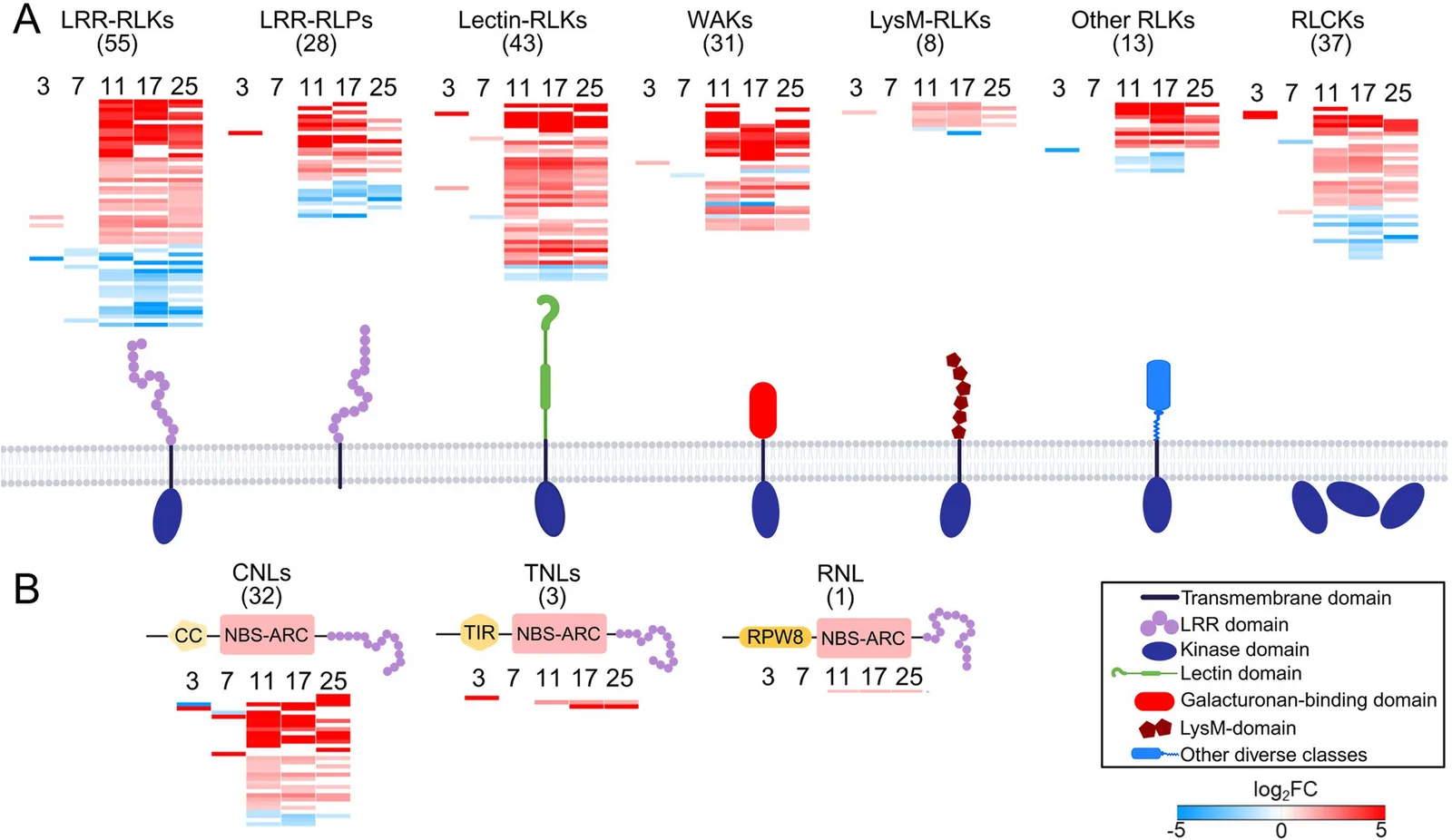
Differential expression of various classes of cell surface receptors, intracellular kinases (**A**), and intracellular nucleotide-binding LRR receptors (NLRs) (**B**). LRR – leucine-rich repeat; RLKs - receptor-like kinases; RLPs – receptor-like proteins; WAKs – Wall-associated kinases; LysM – Lysin motif; RLCKs – receptor-like cytoplasmic kinases; CNLs - CC (coiled-coil) domain nucleotide-binding (NB) LRR receptors (NLRs); TNLs - TIR (Toll/interleukin-1 receptor) domain NLRs; RNLs - RPW8 (resistance to powdery mildew 8)-like coiled-coil domain NLRs

Functional categorization indicated that defense response was the most altered biological process during *C. beticola* infection. Collectively, 1,136 DEGs (19% of the total number of DEGs) were related to the defense response, including DEGs categorized under defense signaling, hypersensitive response (HR) and programmed cell death (PCD) (Supplementary Table S3; Supplementary Fig. S3-S5) and most occur during symptomatic phase. GO enrichment analysis of these DEGs indicated that the GO term cell surface receptor signaling was consistently enriched among the upregulated genes across all time points. Therefore, to better understand the components of cell surface receptor signaling in sugar beet, we identified and categorized different classes of cell surface pattern recognition receptors (PRRs) using Blast2GO annotation as well as predicted domains and sub-cellular localization (Fig. 5A, Supplementary Table S5).

#### Cell surface receptors are differentially expressed during C. beticola infection

We identified 55 DEGs as putative LRR-receptor-like kinases (LRR-RLKs) based on the presence of predicted leucine-rich repeat (LRR) (IPR032675), transmembrane (TM) helices, kinase domain (IPR000719), as well as putative localization to the cell membrane. Furthermore, 28 DEGs with the same predicted domains and localization that lacked a kinase domain were considered as putative LRR-receptor-like proteins (LRR-RLPs) (Fig. 5A; Supplementary Table S5). Receptors with carbohydrate-binding lectin domains known as lectin-RLKs (LecRLKs) were one of the largest groups of upregulated RLKs. Based on Blast2GO annotation and presence of the kinase domain, we identified 43 LecRLKs in total (Fig. 5A, Supplementary Table S5).

We identified 31 putative wall-associated kinases (WAKs) (IPR025287; IPR032872) and 8 homologs of LysM-RLKs (IPR036779; IPR018392) (Fig. 5A, Supplementary Table S5). Additionally, 13 other DEGs annotated as RLKs were predominantly homologs of cysteine-rich RLKs. Putative receptor-like cytoplasmic kinases (RLCKs) (37 DEGs) were identified based on the predicted kinase domain and localization to the cell membrane or cytoplasm, but without any predicted TM helices (Fig. 5A; Supplementary Table S5). Differential expression of multiple homologs of well-established RLCKs, such as AvrPphB susceptible protein 1 (PBS1), PBS1-like proteins (PBLs), non-functional pseudokinase (ZED1), ZED1-related kinases (ZRK), Protein PTI-compromised receptor-like cytoplasmic kinase 1 (PCRK1), and Protein ROP binding protein kinase 2 (RBK2), were observed (Supplementary Table S5).

#### Intracellular receptors are modulated during C. beticola infection

We identified 69 sugar beet homologs of putative nucleotide-binding (NB) LRR receptors (NLRs) (Fig. 5B, Supplementary information Table S5), based on the presence of NB-ARC (IPR002182), disease resistance, N-terminal (IPR041118), and LRR (IPR032675) domains. Classification by their N-terminal domain placed these NLRs into three anciently diverged classes: coiled-coil domain NLRs (CNLs), the Toll/interleukin-1 receptor domain NLRs (TNLs), and the resistance to powdery mildew 8 (RPW8)-like coiled-coil domain NLRs (RNLs). Among these putative NLRs, we identified 32 DEGs as CNLs which represented the predominant class of differentially expressed NLRs. While few NLRs showed upregulation during the asymptomatic phase, most NLRs were upregulated during the later time points (Fig. 5B; Supplementary Table S5).

### PTI and ETI – related genes are modulated during Cercospora beticola infection

**Fig. 6:**
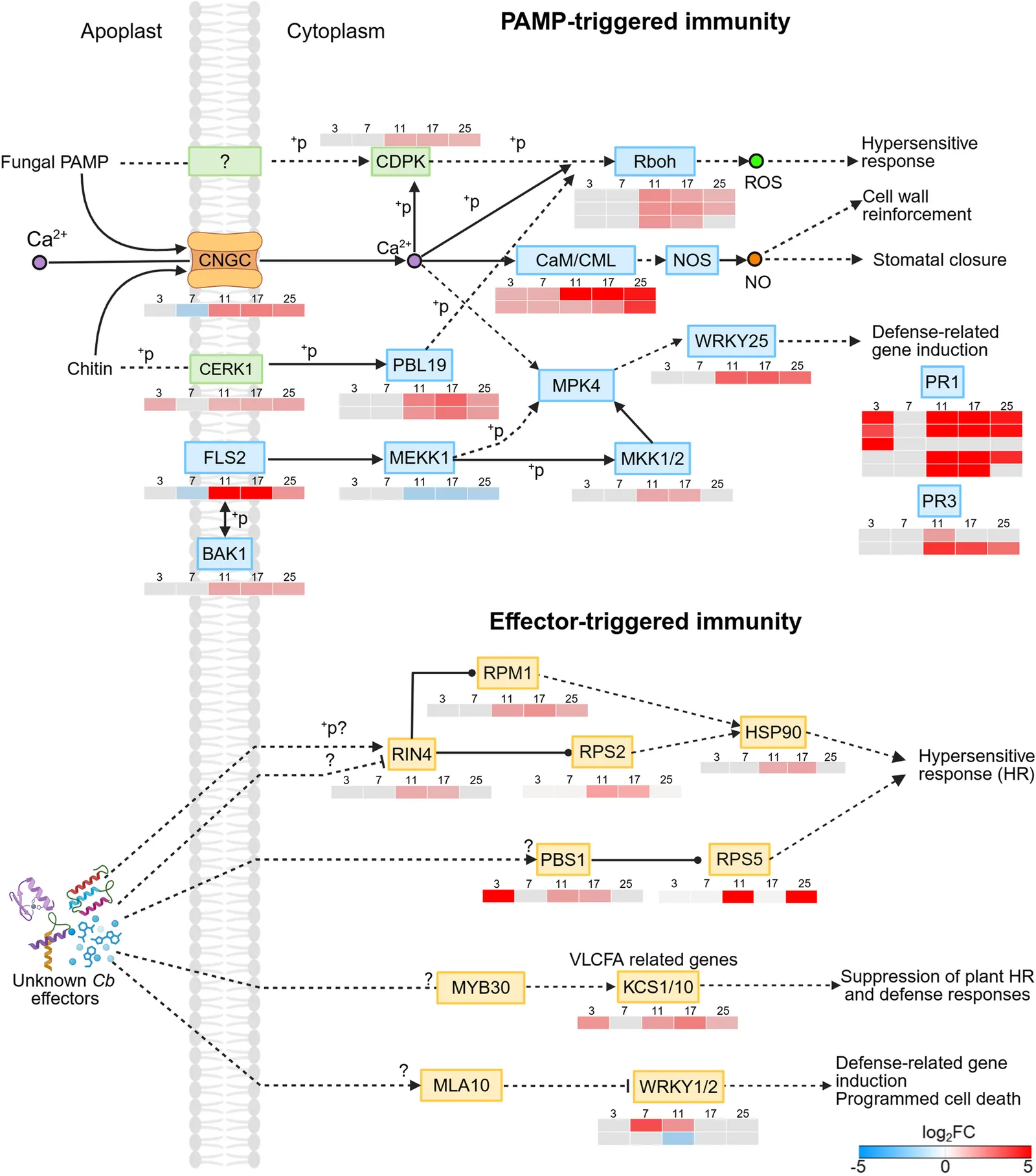
Overview of plant immune signaling pathways and gene expression dynamics. DEGs were mapped onto the plant-pathogen interaction KEGG pathway. Core components of PAMP-triggered Immunity (PTI): Differential expression of pattern recognition receptors (PRRs) such as FLS2 and CERK1 was observed, together with downstream signaling components including RLK (BAK1), RLCK (PBL), MAP kinases (MEKK1, MPK4), cyclic nucleotide-gated channels (CNGCs), calcium sensors (CaM/CML), and respiratory burst oxidase homologs (RBOHs) involved in the production of reactive oxygen species (ROS). These cascades coverage on transcription factors (WRKY25) and regulate defense-related gene expression (PR1 and PR3). Effector-triggered immunity (ETI): Upregulation of putative sugar beet homologs of well-established effector targets (RIN4, PBS1) and their corresponding guard NLRs (RPM1, RPS2, RPS5) was observed, particularly at late points, leading to potential HR. Differential expression of other potential effector targets (KCS1/10, WRKY1/2) was observed in some of the time points during CLS infection

Genes associated with core defense mechanisms of PTI and ETI, such as Ca^2+^ homeostasis, reactive oxygen species (ROS) generation, MAPK activation, callose deposition, regulation of defense-associated transcription factors and defense genes, stomatal closure, and the HR, were differentially expressed throughout the infection time course (Fig. 6). Multiple isoforms of ion channels directly involved in calcium influx and homeostasis were upregulated during the symptomatic phase (Supplementary Table S3). Notably, a CSC1-like protein with calcium-activated cation channel activity was strongly upregulated at 3 dpi (FC: 25.4) and again at 11 dpi (FC: 10.0). Multiple homologs of cell wall-associated peroxidases (PODs) involved in ROS generation were upregulated, especially at time points 11 through 25 dpi. All three identified defense-associated MAPKs were upregulated during the symptomatic phase. Among 36 DEGs with GO annotations related to callose deposition, callose synthase isoform 11 was exclusively downregulated at 3 dpi. Moreover, most of the DEGs associated with callose deposition were upregulated during the symptomatic phase (Supplementary Table S3).

Several major families of defense-related transcription factors (TFs) were differentially expressed, including bHLH (basic helix-loop-helix) (7 DEGs), bZIP (basic leucine zipper) (3 DEGs), AP2/ERF (APETALA2/ethylene responsive factor) (22 DEGs), MYB (myeloblastosis-related) (13 DEGs), NAC (NAM, ATAF and CUC) (6 DEGs), and WRKY (18 DEGs). While most of these TFs showed strong upregulation during 11 to 25 dpi, some of the ERFs and WRKY TFs also showed upregulation at early time points.

Among the DEGs related to stomatal movement, we observed upregulation of a stomatal closure-related actin-binding protein and a guanine nucleotide-binding protein (FC: 9.0) at 3 dpi. While at 7 dpi, the same homolog of guanine nucleotide-binding protein was significantly downregulated (FC: -9.8). During the symptomatic phase, 21 genes associated with stomatal movement were consistently upregulated, including multiple homologs of flavone 3’-O-methyltransferases, different classes of ion channels, transporters, and a wide array of receptor kinases associated with stomatal movement.

Several defense‑related genes considered as markers of defense induction were upregulated at 3, 11, 17, and 25 dpi. These included SAR-associated pathogenesis-related (PR) proteins (Fig. 6; Supplementary Fig. S4), isoforms of chitinases, endochitinases, glucan endo-1,3-beta-glucosidases, beta-D-xylosidases, thaumatin-like proteins, pectinesterase inhibitors, and osmotin-like proteins. At 3 dpi, 16 SAR-associated genes and PR proteins were induced (FC: 1.4 to 8.8), with 10 also upregulated during the symptomatic phase. Furthermore, 25 SAR-related genes were consistently upregulated during the symptomatic phase (Supplementary Fig. S4).

**Fig. 7:**
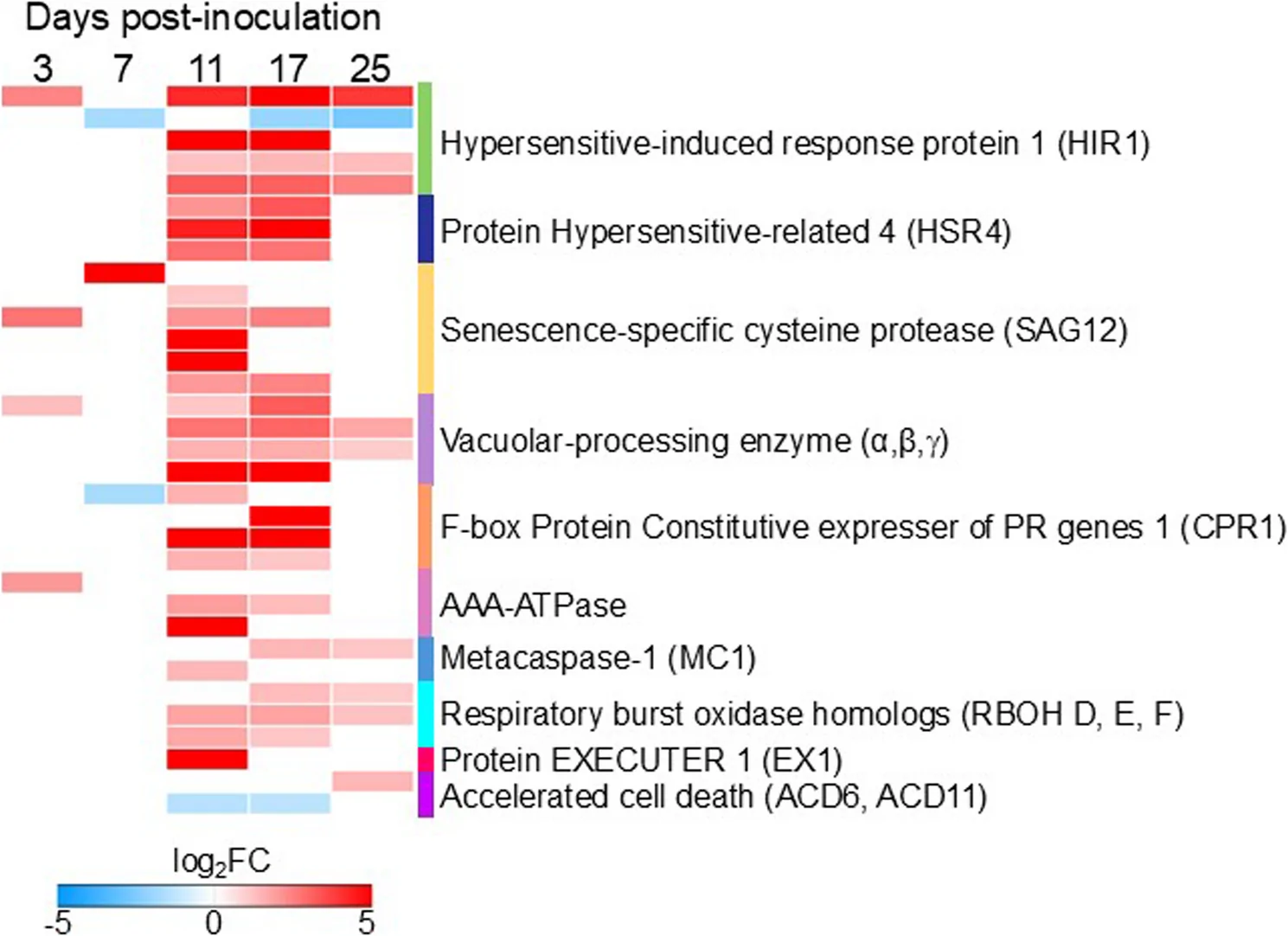
Differential expression of hypersensitive response and programmed cell death–related genes

Among DEGs related to HR and/or PCD, we identified multiple homologs of functionally characterized genes (Fig. 7; Supplementary Fig. S5). Notably, eight DEGs, including homologs of HIR1 (FC: 2.4) and SAG 12 (FC: 2.7) directly associated with HR and PCD, were upregulated at 3 dpi. While at 7 dpi, most of the HR- and PCD-related DEGs were predominantly downregulated, including a flavin-containing monooxygenase (FC: -8.1). The majority of HR and PCD-related genes showed strong upregulation during the symptomatic phase. Downregulation of some homologs of phospholipases, flavin-containing monooxygenases, cysteine proteases, catalase-2, and multiple auxin response factors with the GO annotation “cell death” were also observed during this phase. Furthermore, based on the framework established by Bjornson et al., [5] as core immunity response (CIR) genes in *A. thaliana* in the context of PTI, we identified 29 putative sugar beet homologs of CIR genes (Supplementary Table S6).

### Putative sugar beet homologs of NLRs and guardees are differentially expressed during infection

Given the differential expression of numerous putative sugar beet NLR homologs, we examined the expression of homologs of known effector targets guarded or monitored by NLRs.

#### Guarding protease-cleaved kinases: PBS1 - RPS5 module

RPS5 is reported to guard PBS1, an RLCK that is cleaved by the *Pseudomonas syringae* effector AvrPphB [47]. There was strong upregulation of a putative sugar beet homolog of CNL RPS5, exclusively at 11 and 25 dpi (Fig. 6; Supplementary Table S7). We identified a sugar beet homolog of PBS1 that was exclusively induced (FC: 7.39) at 3 dpi, while the other was moderately upregulated at time points 11 and 17 dpi (Fig. 6; Supplementary Table S7).

#### Monitoring modifications of kinases: ZAR1-ZRK-PBL network

*Arabidopsis thaliana* ZAR1 is a CNL that senses various effector proteins by associating with homologous pseudokinases called ZRKs (ZED1-related kinases) and a class of RLCKs called PBLs (PBS1-like) [30, 58]. We observed upregulation of a sugar beet homolog of ZAR1 (FC: 8.2) at 25 dpi, and differential expression of multiple homologs of ZED1, ZRKs, and PBLs through the infection cycle (Supplementary Table S7).

#### Sensing host protein perturbation: RIN4-RPM1-RPS2 module

Plasma membrane-localized RIN4 (RPM1-interacting protein 4) is a multi-effector target that is guarded by the NLRs RPM1 and RPS2 [52]. One sugar beet homolog of RIN4, predicted to localize to the cell membrane, was moderately upregulated at 11 and 17 dpi. Correspondingly, the sugar beet homolog of RPS2 was exclusively upregulated at the same time points, while the RPM1 homolog was upregulated during the symptomatic phase (Fig. 6; Supplementary Table S7).

Additionally, by mapping DEGs onto the plant-pathogen interaction pathway, we identified putative sugar beet homologs of other known effector targets such as 3-ketoacyl-CoA synthase (KCS) and WRKY1/2 transcription factors (Fig. 6).

### Diverse stress response-related genes are transcriptionally reprogrammed during C. beticola infection

We noticed many DEGs related to oxidative stress, salt stress, and osmotic stress. In total, 122 DEGs were identified as potential general stress response (GSR)-related genes based on homology to *A. thaliana* genes reported as upregulated in response to various elicitors as well as abiotic stress treatments [5] (Supplementary Table S6). Correspondingly, several genes directly associated with stress mitigation and redox homeostasis encompassing a wide range of enzymes involved in detoxification, peroxide reduction, and ROS scavenging were differentially expressed (Fig. 3; Supplementary Table S3; Fig. S2A).

### Hormone-related genes are modulated during C. beticola infection

The second most abundant category of DEGs were associated with hormone-related processes (Fig. 3; Supplementary Table S3; Supplementary Fig. S6). The majority of DEGs related to phytohormones were observed during symptom development. Genes directly related to biosynthesis, metabolism, and transport of hormones as well as genes responsive to hormones were identified using GO annotations. Several DEGs were related and responsive to more than one hormone. Collectively, the highest number of hormone-related DEGs were related to abscisic acid (ABA; 410 DEGs), followed by auxin (IAA; 251 DEGs), jasmonic acid (JA; 208 DEGs), salicylic acid (SA; 200 DEGs), and ethylene (ET; 146 DEGs) (Supplementary Fig. S6). A relatively smaller subset of DEGs related to gibberellic acid (GA; 101 DEGs), brassinosteroid (BR; 93 DEGs), and cytokinin (CT; 81DEGs) were also identified (Supplementary Fig. S6).

### Promoter enrichment identifies WRKY-AHL as a potential regulator of defense in the late stages of C. beticola infection

Cis-element enrichment analysis revealed a significant overrepresentation of defense-related WRKY and AT-hook motif nuclear-localized protein (AHL) binding motifs within the protomer regions of genes upregulated from 11 to 25 dpi (Supplementary Table S8). Among these, 37 distinct WRKY and AHL motifs were consistently enriched across all three time points, with WRKY57 and AHL13 motifs being the most significant within their respective families.

To further explore the proportion of downstream targets of WRKY57 and AHL13 motifs, a FIMO-based motif search was mapped against the functional GO categories (Supplementary Table S8). A significant proportion of defense and hormone-related DEGs were identified to harbor both the WRKY57 and AHL13 binding motifs. These data strongly suggest a coordinated, dual-family regulatory architecture where WRKY transcription factors and AHL proteins potentially act as a master regulatory hub modulating the host defense response during late-stage infection.

### Phytohormone levels are temporally regulated during C. beticola infection

**Fig. 8:**
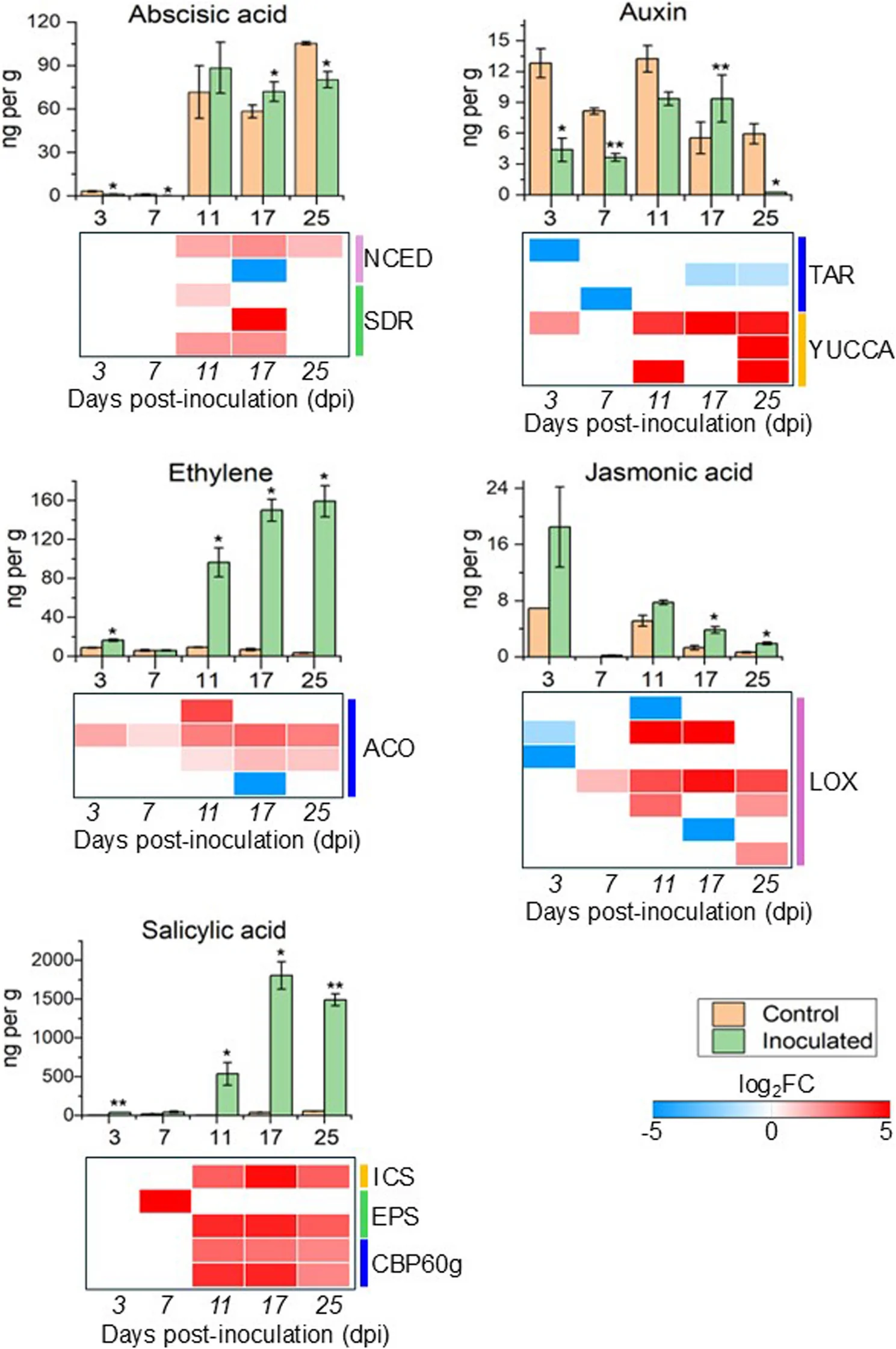
Quantification of hormone content in sugar beet leaves infected with *C. beticola* by LC-MS/MS. * and ** indicate significant difference at *p* value < 0.05 and *p* value 0.01, respectively. Heatmaps display the RNA-seq-based expression profiles of genes encoding rate-limiting enzymes and key biosynthetic genes for each hormone. Abscisic acid (NCED: 9-cis-epoxycarotenoid dioxygenase; SDR: short-chain dehydrogenase reductase); Auxin (TAR: tryptophan aminotransferase; YUCCA: indole-3-pyruvate monooxygenase); Ethylene (ACO: 1-aminocyclopropane-1-carboxylate oxidase); Jasmonic acid (LOX: Lipoxygenase); Salicylic acid (ICS: isochorismate synthase; EPS: ENHANCED PSEUDOMONAS SUSCEPTIBILTY 1; CBP60g: calmodulin-binding protein 60 G)

Considering that a large proportion of hormone-related genes were differentially expressed, we quantified ABA, IAA, JA, SA and 1-aminocyclopropane-1-carboxylate (ACC), the immediate precursor of ET, in the same leaf samples from which RNA was sourced (Fig. 8). We observed reduced levels of ABA and IAA during the asymptomatic phase compared to their respective control samples. While there was an increase at 17 dpi, significantly reduced levels were noticed at 25 dpi for both phytohormones. Relatively high levels of ET (ACC) and SA were evidenced at 3 dpi, and an exponential increase was observed during the symptomatic phase. Significantly increased JA content was observed at 17 and 25 dpi compared to uninoculated controls. We observed similar trends between hormone levels and expression of a few key hormone biosynthetic genes (Fig. 8).

## Discussion

Previous transcriptomic studies on CLS have primarily contrasted the responses of resistant and susceptible genotypes [24, 33, 65]. Despite these advancements, the molecular responses modulated in a susceptible sugar beet genotype during infection with *C. beticola* are still poorly understood. In the present study, we conducted a time-resolved RNA-seq analysis concurrently with hormone profiling of the entire CLS disease cycle, spanning both the asymptomatic and symptomatic phases in a susceptible sugar beet genotype. By focusing exclusively on the susceptible host and sampling across the full infection cycle, our analysis provides a more resolved view of transcriptional dynamics compared with previous transcriptomics studies on CLS [24, 33, 65]. Our functional categorization and enrichment analyses revealed that defense response and hormone-related processes were the most significantly altered responses in sugar beet leaves during infection with *C. beticola*. Significant enrichment of defense-related genes among the differentially expressed sugar beet genes in response to *C. beticola* was reported previously [24, 33, 65]. Similarly, enrichment of KEGG pathways such as plant-pathogen interaction and plant hormone signal transduction were also reported in previous comparative transcriptome studies [24, 33]. Along with these reports, our data suggests a consistent transcriptional modulation of defense and hormone signaling pathways during CLS infection. Our data complement and extend existing transcriptomic studies on the sugar beet-*C. beticola* interaction by unraveling the transcriptional modulation of putative sugar beet PRRs, NLRs, and core defense genes, while incorporating quantitative hormone profiling.

### Early activation, transient suppression, and late reactivation define CLS defense dynamics

For pathogen perception at the cell surface, the plant’s innate immune system relies on PRRs, such as receptor-like kinases (RLKs) and receptor-like proteins (RLPs) [26, 37]. Previously, Weltmeier et al., [65] reported a differentially expressed putative sugar beet RLP with an LRR domain in response to *C. beticola* infection. Based on various in silico analyses, we identified a wide range of genes encoding cell surface receptors (Fig. 5A), and some of the putative cell surface receptors (2 LRR-RLK, 1 LRR-RLP, 2 LecRLKs, 1 LysM-RLK, 1 WAK, and 2 RLCKs) showed upregulation at 3 dpi, our earliest time point in the asymptomatic phase (Fig. 5A; Supplementary Table S5). Similarly, in the early stage of wheat infection by *Zymoseptoria tritici* — an ascomycete fungus that causes septoria tritici blotch and is closely related to *C. beticola —* the most induced genes were RLKs [35]. A significantly higher number of kinases were upregulated in a susceptible potato cultivar compared to a resistant cultivar in response to *Phytophthora infestans* [31].

Along with increased expression of multiple PRRs, we also observed upregulation of putative intracellular NLRs (Fig. 5B; Supplementary Table S5), and genes associated with PTI and ETI defense responses (Fig. 6), including PR proteins, SAR-, HR-, and PCD-related genes at 3 dpi (Fig. 7). HR- and SAR-related defense responses are more often associated with ETI than PTI [12]. An HR-like cell death was observed in a compatible interaction between wheat and *Z. tritici* [27], and upregulation of several senescence-associated genes (SAGs) was reported during the early stages of infection [35]. Recent research established that there is a synergistic relationship between PTI and ETI, wherein they are functionally interdependent and mutually reinforcing [68]. Considering this, upregulation of multiple PRRs and PTI-associated genes could have triggered the upregulation of genes associated with the ETI response (NLRs, HR-, PCD-, and SAR-associated genes) that were observed at 3 dpi. It is also possible that the NLR-based intracellular defense system of sugar beet is transcriptionally activated upon perceiving *C. beticola* effectors as early as 3 dpi. The strong upregulation of putative sugar beet homologs of PBS1 and ZED1, well-established targets of multiple effectors [8], further supports this notion (Supplementary Table S7).

Phytopathogens secrete effectors to suppress, modulate, subvert, and exploit host defense and metabolism to ultimately promote colonization of the host plants [32, 49]. Despite the potential transcriptional activation of PTI and ETI defense responses at 3 dpi, we also observed early downregulation of a subset of PRRs, NLRs, and a few genes associated with certain defense responses (callose synthase isoform 11 involved in callose deposition). Enrichment of GO terms such as innate immune response and cell death among the downregulated genes at 3 dpi may suggest that some specific defense responses are modulated by *C. beticola* at this infection stage. Previously, no significant defense response was observed in the early to mid-stages of infection in polygenic resistant or susceptible sugar beet genotypes at the transcriptome level, speculated to be due to dampening of defense responses by *C. beticola* [65]. Similarly, downregulation of wheat SAGs during the early latent infection stage was attributed to *Z. tritici* delaying and regulating the senescence process [35]. Downregulation of host defense response was evident in our study at 7 dpi. For instance, a guanine nucleotide-binding protein classified as a putative CIR gene was strongly upregulated at 3 dpi (FC: 9.0) but significantly downregulated at 7 dpi (FC: -9.8) (Supplementary Table S6). Almost all the DEGs classified as cell surface PRRs, intracellular NLRs, SAR- and HR-related genes, and genes associated with the metabolism of defense-related compounds were generally downregulated or showed only a minor increase in gene expression level compared to the DEGs in other time points (Supplementary Fig. S3-S5). It is plausible that this time point represents an infection stage wherein *C. beticola* has largely attenuated host defense responses and begun to reconfigure host physiology and metabolism, which is congruent with other hemibiotrophic fungi that actively suppress host immunity and remodel host metabolism during the early asymptomatic stages of infection before the transition to necrotrophy [32, 43].

In at least two of the three time points representing the symptomatic phase, we observed consistent upregulation of all classes of PRRs (28 LRR-RLKs, 10 LRR-RLPs, 29 LecRLKs, 16 WAKs, 6 LysM-RLKs, and 17 RLCKs) and 38 NLRs (Fig. 5A, B; Supplementary Table S5). Most of the defense-related DEGs were also upregulated during these time points (Supplementary Tables S3, S6; Supplementary Fig. S3-S5). For instance, 26 SAR–related genes and 44 h- and PCD-associated genes showed strong upregulation consistently across the time points 11 to 25 dpi. Putative sugar beet homologs of CIR genes were also predominantly upregulated during this phase, suggesting a possible induction of defense responses [5] (Supplementary Table S6). Furthermore, our cis-element enrichment analysis identified WRKY transcription factors and AHL proteins as potential regulators driving the defense response during the symptomatic phase (Supplementary Table S8). Previously, WRKY transcription factors alongside AHL chromatin-remodeling proteins have been shown to serve as essential hubs for activating broad plant defense response networks [40]. Taken together, we observed a biphasic regulation of defense-related genes. This was more prominent with genes related to defense-signaling, HR, and PCD (Fig. 3; Supplementary Fig. S2A; Supplementary Fig. S5). Interestingly, a similar trend in SAGs and other senescence-related genes was observed in the wheat-*Z. tritici* interaction, where it was postulated that senescence-related processes may be regulated in two waves during *Z. tritici* infection [35]. Previous research has shown that NLR-mediated cell death can be co-opted to enhance disease, particularly with necrotrophic pathogens [16]. Indeed, several of the plant susceptibility genes belong to the NLR class of disease resistance genes (e.g. *LOV1*,* Pc*,* Tsn1*) [15]. Increased expression of a multitude of putative NLRs at 11 dpi coincided with the onset of characteristic CLS symptoms (Fig. 5; Supplementary Table S5). This may suggest that at least some of these NLR-mediated signaling cascades, potentially leading to HR and cell death, may be beneficial or even exploited by *C. beticola* during the symptomatic phase of CLS disease.

### Integrated transcriptomic and hormone profiling reveals coordinated phase-dependent defense regulation during CLS infection

Hormones and hormone-mediated signaling networks govern plant immunity as well as growth, making them key players in mediating host-microbe interactions [22]. Regulation of defense responses against microbial attack by the plant defense hormones SA, JA, and ET is complex and interdependent. Defense against biotrophs is positively regulated by SA, whereas JA/ET act synergistically against necrotrophic pathogens [20]. Therefore, it is highly likely that modulation in levels of these major defense hormones could have been driving the biphasic regulation of defense and hormone-related genes we observed earlier.

In the present study, increased levels of SA were observed at 3 dpi and at 11 to 25 dpi, coinciding with upregulation of SA-biosynthetic genes (Fig. 8). A transient increase in SA levels followed by a decrease was previously observed in sugar beet leaves during CLS disease [24]. SA is primarily involved in regulating plant immune signaling and defense pathways against biotrophs and hemibiotrophs [53]. Upregulation of PR-1, a marker for SA accumulation [17], specifically at these time points corroborates with high SA levels (Fig. 6). Accumulation of SA is tightly associated with activation of HR during resistance gene-mediated defense responses [48]. Hence, high SA levels we observed during the symptomatic phase may be regulating the transcriptional activation of a multitude of putative NLRs (Supplementary Table S5) and genes related to the HR and PCD (Fig. 7).

An array of key processes related to growth, development, and stress are regulated by the lipid-derived hormone JA [19]. Increased JA levels were observed during the late time points of the symptomatic phase (17 and 25 dpi). This suggests that the host may have initiated a canonical necrotroph-oriented defense response [46], upon perceiving extensive necrosis at these time points. In interactions with necrotrophic and hemibiotrophic pathogens, resistant genotypes frequently show a stronger and/or earlier JA accumulation than susceptible genotypes [69]. Although the JA levels were high, a gradually decreasing trend was observed during the symptomatic phase (Fig. 8). The declining levels of JA may be pointing to the inability of the susceptible host genotype to accumulate effective levels of JA during this phase, contributing to its CLS susceptibility. A comparative study by Jia et al., [24] revealed that the highly susceptible sugar beet genotype maintained the lowest JA content across all sampling periods compared to resistant and moderately resistant genotypes and the JA levels decreased as the disease progressed. The antagonistic interaction between SA and JA has been extensively studied [46], and there is substantial evidence to support SA-mediated suppression of JA accumulation and JA-mediated signaling [7]. Hence, the elevated SA levels observed at these time points may have contributed to the suppression of JA accumulation and signaling (Fig. 8). It remains unknown if and how *C. beticola* modulates SA and JA biosynthesis or diverts its precursors to achieve a conducive hormonal and metabolic state of the host to facilitate necrotrophy.

The gaseous phytohormone ET regulates key processes underlying plant growth, development, and defense responses [4]. The role of ET signaling in promoting susceptibility to hemibiotrophic and necrotrophic pathogens is well-documented [59]. Onset of HR and HR-related genes coincided with elevated ET levels [23]. Consistently, increased ET levels at 3, 11, 17, and 25 dpi could be corroborated by the upregulation of HR and PCD-related genes in these time points (Figs. 7 and 8). This suggests that ET along with SA may regulate the activation of HR in sugar beet - *C. beticola* interaction.

The phytohormone ABA regulates core plant developmental processes such as seed maturation, dormancy, photoregulation, senescence, and flowering and has a primary function in response to salt, drought, osmotic, and cold stress [3]. Previously, Schmidt et al., [56] reported that sugar beet ABA levels spiked just after inoculation (0 dpi) and declined at 1–2 dpi in *C. beticola-*inoculated leaves. Subsequently, a significant increase in ABA was detected again at 5 to 9 dpi, and a 3.5 to 5.3-fold increase in ABA levels was reported in partially necrotic leaves at 15 dpi. We observed a consistent trend with the ABA levels, wherein significantly reduced levels of ABA were evidenced in our early time points (3 and 7 dpi). Elevated levels of ABA were noticed at 11 dpi, with a significant increase at 17 dpi, a time point when we observed partially necrotic leaves (Figs. 1 and 8). Although the role of ABA in plant disease resistance is complex and not well defined, there is evidence to support that ABA interferes with plant immunity indirectly [36]. For instance, ABA application caused a strong reduction in sugar beet PAL promoter activity and transcript accumulation [56]. Exogenous ABA treatment was also shown to suppress phytoalexin and lignin synthesis, reduce SA concentration, and transcript levels of JA- and ET‐responsive defense genes, indicating an antagonistic effect of ABA on SA and JA/ET signaling [1, 36, 41]. The lower ABA levels we observed during the asymptomatic phase could function to circumvent the repression of SA and ET - mediated defense responses. Taking into consideration that most of the DEGs related to hormones were associated with ABA (Supplementary Fig. S6), modulation of ABA levels may serve as a regulatory axis steering the SA/JA/ET signaling network and consequently, the observed biphasic trend of defense response in sugar beet-*C. beticola* interaction.

Auxins are a group of indolic molecules that have long been recognized for their multiple important roles in regulating plant growth, development, and host-pathogen interactions [29]. Perception of the PAMP flg22 results in repression of auxin-responsive genes in *Arabidopsis*, and the PTI response has been shown to repress auxin and auxin-mediated signaling [42]. Significantly reduced auxin levels observed during early time points (Fig. 8) may have been a consequence of upregulation of several defense-related genes at these time points and may suggest a shift in physiological prioritization towards defense rather than growth and development. Interactions involving several necrotrophic fungi (e.g., *Botrytis*, *Rhizoctonia*, and *Alternaria*) have been shown to induce auxin levels [29]. Among several other contributing factors, a significantly high auxin level, specifically at 17 dpi in our dataset, may point to an infection stage wherein *C. beticola* may have completely transitioned into a necrotroph. Since the accumulation of SA has been shown to repress auxin signaling [64], the significant decrease in auxin levels we observed at 25 dpi could be partially regulated by high levels of SA accumulation at this time point (Fig. 8). Our study has shed light into the modulation of hormone levels and hormone-related genes in sugar beet during CLS infection. However, it remains to be understood if and how hormones and hormone-mediated signaling pathways in sugar beet are directly manipulated by *C. beticola* to cause CLS disease successfully.

### Conclusion and future perspective

The present study has revealed the global reprogramming of the leaf transcriptome of sugar beet during infection with *C. beticola.* These results provide insight into various classes of putative sugar beet PRRs and NLRs that are modulated at the transcriptome level. Our data showed a wave-like regulation of defense responses, especially the HR in sugar beet during *C. beticola* infection. It will be important to identify nodes of defense and hormone-mediated signaling cascades that are actively suppressed during the asymptomatic infection phase and are specifically co-opted at the late symptomatic stages of infection by *C. beticola*. It will also be important to understand the metabolic state of sugar beet and other cues that *C. beticola* exploits to trigger the transition to a necrotrophic mode of nutrient acquisition. Future efforts should focus on identifying differentially expressed effector genes in *C. beticola* so that they may be functionally characterized for their role in modulating the sugar beet defense response.

## Supplementary Information

Supplementary Material 1. Table S1. Summary of read counts and reads mapped to the sugar beet genome. Table S2. PERMANOVA and beta dispersion tests to support PCA. Table S3. List of DEGs with functional category and annotation. Table S4. List of statistically significant gene ontology terms for up- and down-regulated genes at each time point. Table S5. List of putative cell surface and intracellular receptors. Table S6. List of putative homologs of GSR and CIR genes. Table S7. List of putative homologs of NLRs and their guardees. Table S8. Cis-element enrichment analysis of DEGs.

## Data Availability

Transcriptome data used for the analysis have been deposited in the NCBI SRA database as Bioproject PRJNA1433507 (Accession number: SUB16041308). All other data generated or analyzed in this study are included as supplementary information files.

## Abbreviations

CLS: Cercospora leaf spot
dpi: days post-inoculation
DEGs: Differentially expressed genes
log_2_FC: log_2_fold-change
GO: Gene Ontology
KEGG: Kyoto Encyclopedia of Genes and Genomes
NLRs: Nucleotide-binding (NB) leucine‑rich repeat (LRR) receptors
PTI: Pattern-triggered immunity
ETI: Effector-triggered immunity
HR: Hypersensitive response
SAR: Systemic acquired resistance
ABA: Abscisic acid
SA: Salicylic acid
JA: Jasmonic acid
ET: Ethylene
IAA: Auxin
